# Wer kann sich vorstellen, von Angehörigen gepflegt zu werden? Ergebnisse einer repräsentativen Befragung 65- bis 75-Jähriger in der Region Bodensee-Oberschwaben

**DOI:** 10.1007/s00391-022-02073-z

**Published:** 2022-05-28

**Authors:** Lea Raiber, Florian Fischer, Claudia Boscher, Johannes Steinle, Maik H. -J. Winter

**Affiliations:** Institut für Gerontologische Versorgungs- und Pflegeforschung, Hochschule Ravensburg-Weingarten, Doggenriedstr., 88250 Weingarten, Deutschland

**Keywords:** Langzeitpflege, Pflegebedürftigkeit, Familiäre Pflege, Häusliche Pflege, Versorgungsforschung, Long-term care, Care dependency, Family care, Home care, Care research

## Abstract

**Hintergrund:**

Die Zahl pflegebedürftiger Personen wird in den kommenden Jahren, bedingt durch den demografischen Wandel, weiterhin zunehmen. Um eine hochwertige bedarfs- und bedürfnisorientierte pflegerische Versorgung zu gewährleisten, nimmt die Berücksichtigung der Pflegepräferenzen eine besondere Bedeutung ein.

**Ziel der Arbeit:**

Dieser Beitrag soll zum einen die Pflegepräferenzen 65- bis 75-Jähriger darstellen und zum anderen Prädiktoren der Präferenz für eine Pflege durch Angehörige identifizieren.

**Methoden:**

Es wurde eine repräsentative Bevölkerungsbefragung der 65- bis 75-Jährigen in der Region Bodensee-Oberschwaben durchgeführt (*n* = 625). Die Daten werden deskriptiv dargestellt und hinsichtlich der Präferenz für informelle Pflege bi- und multivariat (*χ*^2^-Tests; logistische Regression) ausgewertet.

**Ergebnisse:**

Die Mehrheit der Befragten präferiert eine Pflege im häuslichen Umfeld; zumeist mit professioneller Unterstützung. Zudem stehen die Befragten „alternativen“ Wohnformen (z. B. Mehrgenerationenwohnen oder Wohngemeinschaften für ältere Menschen) positiv gegenüber.

Bedeutende Prädiktoren für die Präferenz informeller Pflege sind das männliche Geschlecht, das Vorhandensein weiterer im Haushalt lebender Personen, eine eigene Pflegebereitschaft und ein fehlender Berufsabschluss.

**Diskussion:**

Die Präferenz für eine pflegerische Versorgung im häuslichen Umfeld bringt Herausforderungen für informell und formell Pflegende sowie für die Pflegebedürftigen selbst mit sich, die zu einem Spannungsfeld zwischen den Erwartungen und Möglichkeiten führen können. Zur Sicherstellung einer zukunftsfähigen und nutzer*innenorientierten pflegerischen Versorgung bedarf es daher einer qualitativen sowie quantitativen Weiterentwicklung der ambulanten Versorgung.

## Abstract (en)

**Background:**

The number of people in need of care will continue to increase in the coming years due to demographic change. In order to ensure high-quality and needs-oriented nursing care, preferences related to nursing need to be taken into consideration.

**Objective:**

This paper aims 1) to describe the care preferences related to nursing care of 65–75-year-olds and 2) to identify predictors for the preference to be cared for by relatives.

**Methods:**

A representative population survey of 65–75-year-olds in a region in southern Germany was conducted (*n* = 625). Data are presented descriptively and analyzed bivariately and multivariately (*χ*^2^-tests; logistic regression) with respect to preferences for informal care.

**Results:**

The majority of respondents prefer long-term care at home; for most participants, professional assistance is preferred. In addition, respondents had a positive attitude towards alternative forms of living (e.g., multigenerational living or shared housing for the old).

Significant predictors for the preference of informal care were male gender, the presence of other persons living in the household, willingness to provide care on one’s own, and lack of a professional degree.

**Conclusion:**

The preference to provide care in the home environment poses challenges for informal and formal caregivers as well as for those in need of care themselves, which can lead to tension between expectations and possibilities. In order to ensure sustainable and user-oriented long-term care, it is therefore necessary to further improve quality and quantity of outpatient care.

## Hinführung zum Thema

Aufgrund der in Deutschland bereits hohen und zukünftig noch steigenden Zahl an Pflegebedürftigen ergeben sich Herausforderungen für die professionelle sowie informelle Pflege. Dies geht einher mit einem gesellschaftlichen Wandel, der sich u. a. in veränderten Familienstrukturen und einem Generationenwechsel darstellt sowie Auswirkungen auf Erwartungen an die Lebensgestaltung und pflegerische Versorgung mit sich bringt. Um zukünftig eine hochwertige bedarfs- und bedürfnisorientierte Pflege zu gewährleisten, kommt dem Wissen um Pflegepräferenzen daher eine große Praxisrelevanz zu.

## Hintergrund und Fragestellung

Innerhalb der Gesellschaft zeigen sich individuelle Präferenzen für die pflegerische Versorgung im Alter, die – beispielsweise durch Veränderungen der Familienstrukturen – einem stetigen Wandel unterliegen. Gleichzeitig beinhalten diese heterogenen Vorstellungen bestimmte anhaltende Kernelemente; so wird im Fall einer Pflegebedürftigkeit meist eine Versorgung in der eigenen häuslichen Umgebung favorisiert [12, 13, 16, 18, 21]. Dies entspricht dem im SGB XI (Sozialgesetzbuch) formulierten Grundsatz „ambulant vor stationär“: In Deutschland werden, nahezu äquivalent zu den Zahlen für Baden-Württemberg [23], etwa 80 % der Pflegebedürftigen zu Hause gepflegt, 56 % ausschließlich durch Angehörige und 24 % in Kombination oder alleinig durch ambulante Pflege- und Betreuungsdienste. Jede*r Fünfte wird in einer Pflegeeinrichtung betreut, wobei der Anteil der im Pflegeheim Versorgten erst mit zunehmendem Pflegegrad ansteigt [22].

Eine Pflege im häuslichen Umfeld wird u. a. mit einem eigenständigen Leben in vertrauter Umgebung assoziiert [16]. Unabhängig von Ort und Art der gewünschten Pflege hat der Wunsch nach Individualität und Autonomie eine hohe Bedeutung [14]. Im Hinblick auf die Präferenz für eine Pflege zu Hause zeigen die bisher vorliegenden Studien aus Deutschland unterschiedliche Einflussfaktoren: Hajek et al. berichten, dass Personen mit Pflegeerfahrung, ohne vorliegende Pflegebedürftigkeit und mit gering eingeschätztem Gesundheitszustand häufiger häusliche Pflege präferieren [11]. Einer Studie von Spangenberger et al. zufolge sind das Leben in einer Partnerschaft und das männliche Geschlecht positiv mit einer Präferenz für häusliche Pflege assoziiert [21]. Darüber hinaus verstärkt ein gutes Verhältnis zu den unterstützenden Familienangehörigen den Wunsch nach einer Versorgung im häuslichen Umfeld [16]. Das Vorhandensein oder Fehlen privater familiärer Unterstützungsressourcen beeinflusst demnach ebenso wie Pflegeerfahrung die Präferenz für Ort und Form der pflegerischen Unterstützung.

Da der größte Teil der pflegerischen Versorgung durch Familienmitglieder erbracht wird, gelten diese als „größter Pflegedienst der Nation“. Eine familiäre Pflegetätigkeit geht für pflegende Angehörige häufig u. a. mit hoher Belastung sowie finanziellen und beruflichen Einschränkungen einher [4, 15, 25]. Bereits heute ist zu erkennen, dass sich die Verfügbarkeit des informellen Pflegepotenzials reduziert. Dies hängt mit räumlichen Distanzen, Pluralisierung der Familienstrukturen, längeren Erwerbszeiten im Alter und höheren Frauenerwerbstätigkeitsquoten zusammen [19].

Schmidt konstatiert, dass Pflege zukünftig durch eine ausdifferenzierte Nachfrage geprägt sein wird, die aus den heterogenen Präferenzen der pflegerischen Versorgung in der Bevölkerung entsteht [20]. Um den Herausforderungen in der Langzeitpflege zu begegnen, die Pflege zukunftsfähig auszugestalten und den Erwartungen der Leistungsnutzer*innen gerecht zu werden, ist die Berücksichtigung von Pflegepräferenzen von Bedeutung. Vor diesem Hintergrund sowie mit Blick auf den sich in den kommenden Jahren weiter verstärkenden Fachkräftemangel in der Pflege [3] geht dieser Beitrag den Fragen nach, welche Pflegepräferenzen bei 65- bis 75-Jährigen – als Gruppe potenziell zukünftiger Leistungsnutzer*innen pflegerischer Versorgung – bestehen, und welche Faktoren mit der Präferenz für eine ausschließlich durch Angehörige erbrachte Pflege assoziiert sind.

## Methodik

### Studiendesign

Im Jahr 2019 wurde eine repräsentative Bevölkerungsbefragung in der Region Bodensee-Oberschwaben zur Zukunft der Pflege als schriftlich-postalische Erhebung durchgeführt. Die vorliegende Analyse fokussiert mit den Pflegepräferenzen ein wichtiges Element der gesamten Datenerhebung. Die Statistik „Bevölkerungsfortschreibung“ stellte die Datengrundlage dar, aus der die Anzahl der Einwohner*innen nach Altersgruppe, Geschlecht und Gemeinde (und somit Raumkategorie) erhoben wurden.

Bei der Berechnung der minimalen Stichprobengröße (*n* = 381) nach Häder [10] wurde von einem Anteil von 50 % der Befragten mit hoher Präferenz, bei einer Grundgesamtheit von *n* = 50.000 Personen sowie einer Irrtumswahrscheinlichkeit von 5 % ausgegangen. Unter der Annahme eines Rücklaufs von ca. 15 % umfasst die kalkulierte Stichprobengröße 2500 Personen. Auf Basis von Daten der Einwohnermelderegister aller Gemeinden der Region Bodensee-Oberschwaben (Landkreise Ravensburg, Sigmaringen, Bodenseekreis) wurde eine repräsentative Stichprobe aus der Grundgesamtheit 65- bis 75-Jähriger (2019: 68.959 Personen [24]) gezogen. Zielpersonen aus ländlichen Gemeinden wurden mittels einer Klumpenstichprobe ausgewählt (zweistufiges Ziehungsverfahren: 1. Schritt Zufallsauswahl der Gemeinde mit Schichtungsmerkmal Landkreis, 2. Schritt Zufallsauswahl Adresse), Zielpersonen aus Verdichtungsraum und dessen Randzone wurden auf Basis eines einstufigen Ziehungsverfahrens (proportional geschichtete Zufallsstichprobe) ausgewählt. Von einer Repräsentativität hinsichtlich der personenbezogenen Merkmale Alter, Geschlecht und Wohnort (Raumkategorie) kann dadurch ausgegangen werden.

### Samplebeschreibung

Von 2500 angeschriebenen 65- bis 75-Jährigen haben sich 625 an der Befragung beteiligt; dies entspricht einem Rücklauf von 25,0 %. Die Prüfung auf Konsistenz der Antwortangaben zeigte ein sehr gutes Antwortverhalten der Befragten, sodass lediglich ein Fragebogen aufgrund inkonsistenter Angaben für die Analyse ausgeschlossen werden musste. Der Anteil an Pflegebedürftigen (Selbsteinschätzung) beträgt 5,0 % (*n* = 31). Aufgrund der bereits vorliegenden Exposition mit der eigenen Pflegebedürftigkeit wurde diese Personengruppe aus der Analyse ausgeschlossen, sodass das Sample insgesamt 593 Personen umfasst.

In Tab. 1 sind die soziodemografischen Charakteristika der Befragten dargestellt. Das Durchschnittsalter beträgt 69,2 (± 2,8) Jahre, 46,2 % der Studienteilnehmenden sind männlich. Die durchschnittliche Zahl von Kindern liegt bei 1,9 (± 1,2) und variiert zwischen keinen und 7 Kindern.

**Table 1:** 

	Anteil in % (*n*)/M (SD)
*Alter (n* *=* *591)*	69,2 (± 2,8)
*Geschlecht (n* *=* *593)*	
Männlich	46,2 % (274)
Weiblich	53,8 % (319)
*Höchster beruflicher Abschluss (n* *=* *588)*	
Akademischer Abschluss	29,3 % (172)
Meister*in/Techniker*in bzw. Abschluss an einer Berufs- oder Fachakademie	17,0 % (100)
Beruflich-betriebliche bzw. beruflich-schulische Ausbildung	47,6 % (280)
Kein Berufsabschluss	6,1 % (36)
*Haushaltsgröße (n* *=* *587)*	
Einpersonenhaushalt	17,9 % (105)
Mehrpersonenhaushalt	82,1 % (482)
*Raumkategorie (n* *=* *555)*	
Ländlicher Raum	64,7 % (359)
Randzone	15,1 % (84)
Verdichtungsraum	20,2 % (112)
*Anzahl der Kinder (n* *=* *593)*	1,9 (± 1,2)
Keine Kinder	15,3 % (91)
Ein Kind	18,4 % (109)
Zwei oder mehr Kinder	66,3 % (393)

Abweichungen von der Samplegröße (*n* = 593) ergeben sich aufgrund fehlender Werte

*M* Mittelwert, *SD* Standardabweichung

### Fragebogen

Im Fragebogen wurden sowohl standardisierte als auch für die Befragung entwickelte Instrumente genutzt. Die Pflegepräferenz wurde mithilfe des Items „Welche pflegerische Versorgungsform können Sie sich für Ihre eigene Versorgung vorstellen?“ erfasst. Die Studienteilnehmenden wurden gebeten, 10 Versorgungsformen auf einer 4‑stufigen Skala von „gut vorstellbar“ bis „gar nicht vorstellbar“ zu bewerten. Zur Analyse wurden die Antwortmöglichkeiten „gut vorstellbar“ und „eher vorstellbar“ zu einer hohen und „eher nicht vorstellbar“ und „gar nicht vorstellbar“ zu einer niedrigen Präferenz zusammengefasst.

Als abhängige Variable im Regressionsmodell wurde informelle Pflege im Sinne einer häuslichen Pflege ausschließlich durch Angehörige definiert. Zur Modellentwicklung wurden folgende unabhängige Variablen berücksichtigt: Geschlecht, Alter, Raumkategorie, Anzahl der Kinder, Haushaltsgröße, Berufsabschluss, Wohnsituation, Pflegeerfahrung, Gesundheitszustand, Informationsgrad der pflegerischen Versorgung und Pflegebereitschaft gegenüber den Eltern.

### Auswertung

Zunächst erfolgte eine deskriptive Auswertung der Präferenz für verschiedene Versorgungsformen. Welche Faktoren mit der Präferenz einer Pflege durch Angehörige assoziiert sind, wurde mittels einer logistischen Regression untersucht. Eine erste Variablenreduktion erfolgte anhand einer bivariaten Analyse (*χ*^2^-Tests). Die weiter berücksichtigten Variablen (*p* < 0,25) wurden durch eine schrittweise Rückwärtsselektion für die Aufnahme in das Regressionsmodell ausgewählt. Für die multivariate Analyse wurde eine multiple Imputation mit 15 Iterationen durchgeführt, um für fehlende Werte Schätzwerte einzusetzen, welche durch die Verteilung sämtlicher anderer Variablen als Prädiktoren vorhergesagt und durch Zufallsfehler ergänzt wurden. Der Anteil an „missings“ bei den unabhängigen Variablen lag zwischen 0 % (für Haushaltsgröße und Anzahl der Kinder) und 6,1 % (für Raumkategorie) und einer Missing-Quote von 18,3 % bei den in das Regressionsmodell eingeschlossenen Variablen. Der MCAR-Test von Little [17] zeigte auf, dass die Werte nicht vollständig zufällig fehlen. Es wird jedoch von einem zumindest zufälligen Fehlen der Werte („missing at random“) ausgegangen, bei dem die Wahrscheinlichkeit für das Fehlen eines Wertes von einem anderen Merkmal abhängt, aber nicht von der Ausprägung des fehlenden Merkmals selbst. Daher wurden zur Imputation der fehlenden Werte alle erhobenen Variablen genutzt. Dies erfolgte über eine regressionsbasierte multiple Imputation ursprünglich fehlender Werte via „chained equations“. Eine Multikollinearität der Variablen im Modell besteht nicht. Die Analysen wurden mit Stata 16.0 durchgeführt und das Signifikanzniveau auf 5 % festgelegt.

## Ergebnisse

### Pflegepräferenzen

**Figure 1:**
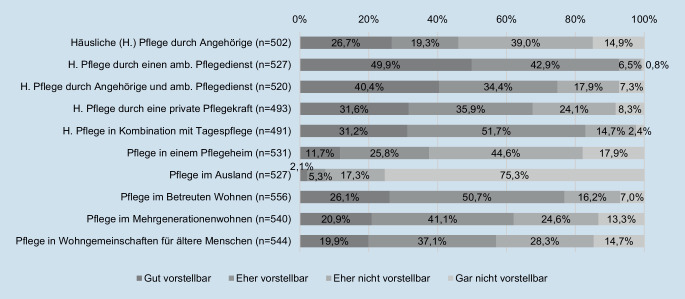

Insgesamt zeigt sich deutlich, dass eine Pflege im vertrauten häuslichen Umfeld präferiert wird (Abb. 1). Trotzdem sind Unterschiede zwischen den Versorgungsformen zu erkennen, denn für 92,8 % ist eine Versorgung durch einen ambulanten Pflegedienst (gut/eher) vorstellbar, während eine Pflege ausschließlich durch Angehörige lediglich von 46,0 % der Befragten präferiert wird.

Für einen weitaus geringeren Anteil entspricht eine Versorgung im Pflegeheim den Vorstellungen. Eine Pflege im Ausland ist für die Mehrheit der Befragten (92,6 %) eher nicht oder gar nicht vorstellbar. Wiederum aufgeschlossen scheinen die 65- bis 75-Jährigen gegenüber einer Pflege in „alternativen“ Wohnformen, beispielsweise Pflege im betreuten Wohnen oder Mehrgenerationenwohnen.

### Prädiktoren für die Präferenz informeller Pflege

Bivariate Assoziationen zwischen Präferenzen für eine Pflege durch Angehörige und erklärenden Variablen zeigen, dass Geschlecht, Haushaltsgröße und Pflegebereitschaft für die eigenen Eltern signifikant mit der Präferenz einer Pflege durch Angehörige zusammenhängen (Tab. 2).

**Table 2:** 

	Niedrige Präferenz^a^	Hohe Präferenz^b^	*p*-Wert^c^
	Anteil in % (*n*)	Anteil in % (*n*)	
Gesamt (*n* = 502)	54,0 % (271)	46,0 % (231)	–
*Geschlecht (n* *=* *502)*			
Männlich	47,4 % (110)	52,6 % (122)	**0,006**
Weiblich	59,6 % (161)	40,4 % (109)	
*Alter (n* *=* *501)*			
Jüngere Altersgruppe (65 bis 69 Jahre)	50,4 % (138)	49,6 % (136)	0,082
Ältere Altersgruppe (70 bis 75 Jahre)	58,1 % (270)	41,9 % (95)	
*Raumkategorie (n* *=* *475)*			
Ländlicher Raum	55,6 % (173)	44,4 % (138)	0,303
Randzone	46,3 % (31)	53,7 % (36)	
Verdichtungsraum	57,7 % (56)	42,3 % (41)	
*Höchster Berufsabschluss (n* *=* *499)*			
Kein Berufsabschluss	33,3 % (10)	66,7 % (20)	0,119
Beruflich-betriebliche bzw. beruflich-schulische Ausbildung	56,1 % (134)	43,9 % (105)	
Meister*in/Techniker*in bzw. Abschluss an einer Berufs- oder Fachakademie	53,5 % (46)	46,5 % (40)	
Akademischer Abschluss	56,3 % (81)	43,8 % (63)	
*Wohnsituation (n* *=* *500)*			
Eigenheim/Eigentumswohnung	52,8 % (210)	47,2 % (188)	0,388
Angemietetes Haus/Mietwohnung	60,4 % (58)	39,6 % (38)	
Mehrgenerationenhaus/betreutes Wohnen^d^	0,0 % (0)	100,0 % (1)	–
Kostenfrei bei/mit Familienangehörigen^d^	40,0 % (2)	60,0 % (3)	–
*Haushaltsgröße (n* *=* *497)*			
Mehrpersonenhaushalt	48,4 % (196)	51,6 % (209)	**<0,001**
Einpersonenhaushalt	78,3 % (72)	21,7 % (20)	
*Anzahl an Kindern (n* *=* *502)*			
Keine Kinder	54,3 % (38)	45,7 % (32)	0,055
Ein Kind	59,1 % (52)	40,9 % (36)	
Zwei oder mehr Kinder	52,6 % (181)	47,4 % (163)	
*Eigene Beteiligung an Pflege (n* *=* *489)*			
Nein, keine Beteiligung	55,6 % (140)	44,4 % (112)	0,418
Ja, unterstützend oder hauptverantwortlich	51,6 % (123)	48,1 % (114)	
*Gesundheitszustand (n* *=* *501)*			
Ausgezeichnet/sehr gut	54,8 % (92)	45,2 % (76)	0,964
Gut	53,5 % (144)	46,5 % (125)	
Weniger gut/schlecht	54,7 % (35)	45,3 % (29)	
*Gefühlter Informationsgrad zur pflegerischen Versorgung (n* *=* *498)*			
Gut/eher gut	52,5 % (181)	47,5 % (164)	0,238
Eher schlecht/schlecht	58,2 % (89)	41,8 % (64)	
*Pflegebereitschaft für die eigenen Eltern (n* *=* *493)*			
Hohe Zustimmung	48,6 % (158)	51,4 % (167)	**<0,001**
Geringe Zustimmung	65,5 % (110)	34,5 % (58)	

^a^Hohe Präferenz ≙ Pflege durch Angehörige gut bzw. eher vorstellbar

^b^Niedrige Präferenz ≙ Pflege durch Angehörige eher nicht bzw. gar nicht vorstellbar

^c^*χ*^2^-Test

^d^Diese Ausprägungen wurden aufgrund der geringen Fallzahl aus der Analyse ausgeschlossen

In Tab. 3 sind die Ergebnisse der logistischen Regression dargestellt. Im Gegensatz zu Frauen favorisieren Männer häufiger eine pflegerische Versorgung ausschließlich durch Angehörige (OR = 1,90, 95 %-KI: 1,27–2,86). Personen in einem Mehrpersonenhaushalt haben, im Vergleich zu alleinlebenden Personen, eine höhere Präferenz für eine Pflege durch Angehörige (OR = 3,05, 95 %-KI: 1,77–5,26). Zudem zeigt sich, dass Personen ohne Berufsabschluss diese Pflegeform im Vergleich zu Akademiker*innen eher präferieren (OR = 3,67, 95 %-KI: 1,50–8,99). Ebenso ist die eigene Bereitschaft zur Pflege der Eltern positiv mit der Präferenz einer familiären Pflege assoziiert (OR = 1,80, 95 %-KI: 1,21–2,67).

**Table 3:** 

	OR	95 %-KI	*p*-Wert
*Geschlecht (Ref. weiblich)*			
Männlich	1,90	1,27–2,86	**0,002**
*Haushaltsgröße (Ref. Einpersonenhaushalt)*			
Mehrpersonenhaushalt	3,05	1,77–5,26	**<** **0,001**
*Höchster Berufsabschluss (Ref. akademischer Abschluss)*			
Meister*in/Techniker*in bzw. Abschluss an einer Berufs- oder Fachakademie	1,09	0,63–1,88	0,755
Beruflich-betriebliche bzw. beruflich-schulische Ausbildung	1,31	0,83–2,06	0,238
Kein Berufsabschluss	3,67	1,50–8,99	**0,004**
*Pflegebereitschaft für die eigenen Eltern (Ref. geringe Zustimmung)*			
Hohe Zustimmung	1,80	1,21–2,67	**0,004**
*Konstante*	0,14	0,07–0,29	**<** **0,001**

Ausgeschlossene Variablen in der Modellentwicklung: Alter, Raumkategorie, Wohnsituation, Anzahl an Kindern, eigene Beteiligung an Pflege, Gesundheitszustand, gefühlter Informationsgrad zur pflegerischen Versorgung

*OR* Odds Ratio, *KI* Konfidenzintervall

## Diskussion

Die Ergebnisse bestätigen vorangegangene Studien, die eine Präferenz für eine pflegerische Versorgung im häuslichen Umfeld von den Befragten aufzeigen [12, 13]. Dies geht mit einer hohen Ablehnung gegenüber einer Pflege im Pflegeheim einher [13, 14]. So ist das Image von Pflegeheimen in der Bevölkerung häufig negativ ausgeprägt, da diese u. a. als „letzte Station vor dem Tod“ [14] bzw. „Institutionen des Sterbens“ [26] gesehen werden. Demgegenüber lässt sich die Präferenz für ein Pflegearrangement im häuslichen Umfeld in erster Linie auf den Wunsch nach Autonomie und Individualität [14] sowie das Gefühl von Vertrautheit und Sicherheit zurückführen [16].

Aus einer Präferenz für informelle Pflege kann sich ein Spannungsfeld zwischen den Wünschen sowie Erwartungen (potenziell zukünftig) Pflegebedürftiger und den Möglichkeiten der Angehörigen ergeben [7]. Ein Mehrpersonenhaushalt weist auf ein höheres informelles Pflegepotenzial hin. Informelle Pflege geht jedoch für die Pflegeperson vielfach mit Einschränkungen des eigenen (Erwerbs‑)Lebens einher [5]. Traditionelle Rollenverständnisse sowie eine höhere Lebenserwartung von Frauen, die zudem vielfach jünger als ihr Lebenspartner sind, tragen dazu bei, dass Frauen immer noch den weitaus größten Teil informeller Pflege leisten [8]. Möglicherweise lässt sich die niedrigere Präferenz von Frauen für eine Pflege durch Angehörige teils durch selbsterlebte Belastungen ihrer bereits geleisteten Pflegearbeit erklären. Denkbar ist auch, dass die niedrigere Präferenz von Frauen für eine Pflege durch Angehörige eine durchaus realistische Zukunftserwartung abbildet, sofern diese aufgrund eines zumeist älteren Lebenspartners eine geringere Wahrscheinlichkeit für partnerschaftliche Pflege haben.

Unsere Studie zeigt zudem auf, dass die Wahrscheinlichkeit zur Präferenz einer Versorgung durch Angehörige höher bei jenen Befragten ist, die selbst ihre Eltern pflegen würden bzw. gepflegt haben. Dies deutet darauf hin, dass Pflege eine moralische Wertigkeit hat und ein Selbstverständnis von bzw. für Pflege durch Angehörige aufgrund des Prinzips intergenerationeller Reziprozität besteht [9]. Ein weiterer Grund für die Übernahme informeller Pflege kann zudem die emotionale Bindung zwischen der pflegenden Person und der*dem Pflegebedürftigen darstellen [15]. Eine Erklärung für die höhere Präferenz informeller Pflege bei Befragten ohne Berufsabschluss könnte darin liegen, dass einkommensschwache Pflegebedürftige den Eigenanteil für Pflegekosten nicht tragen können. Unter dem Druck zur Vermeidung von Zuzahlungen wäre die Wahl der pflegerischen Versorgung somit nicht frei, sondern einkommensabhängig [5].

Um den Präferenzen hinsichtlich einer häuslichen Pflege gerecht zu werden, bedarf es somit einer qualitativen sowie quantitativen Weiterentwicklung der ambulanten Versorgung. Dafür sind Rahmenbedingungen zu schaffen, die eine informelle Pflege – auch unter Einbezug professioneller Unterstützung – ermöglichen, sofern dies sowohl von Pflegebedürftigen als auch Angehörigen die präferierte Versorgungsform darstellt. Hierzu sind Möglichkeiten auf politischer und auch betrieblicher Ebene zur Verbindung von Erwerbsleben und Pflegetätigkeit zu etablieren [2, 27]. Darüber hinaus muss eine flexible Gestaltung der Pflege im häuslichen Setting ermöglicht werden [1]: Dafür sollte u. a. ein Anspruch auf Pflegeleistungen (beispielsweise Geld- und Sachleistungen) unabhängig vom Ort der erbrachten Pflege bestehen. Ebenso erscheint eine weitere Stärkung der Kurzzeitpflege, aber auch der Tages- und Nachtpflege sinnvoll, um Entlastungsmöglichkeiten im Rahmen der informellen Pflege zu schaffen.

Zugleich ist eine umfassende Beratung von Pflegebedürftigen und deren Angehörigen notwendig, um den Bekanntheitsgrad bereits bestehender und ggf. zukünftig eingeführter Leistungsmaßnahmen zu steigern sowie deren Inanspruchnahme zu erhöhen [6]. An dieser Stelle kann auf die bereits etablierte Pflegeberatung nach § 7a SGB XI zurückgegriffen werden, als eine Art Lotsenfunktion im Pflegesystem. Jedoch erscheint eine stärke Ausrichtung der Pflegeberatung auf eine proaktive Ansprache der betroffenen Personen sinnvoll, um frühzeitig aufzuklären und Informationsdefizite zu reduzieren.

### Limitationen

Die Ergebnisse dieser Studie stellen die Sichtweise von 65- bis 75-Jährigen dar und stehen stellvertretend für diese Altersgruppe. Die Erhebung war regional begrenzt, sodass die Befunde nur bedingt verallgemeinert werden können. Eine Verzerrung durch selektive Teilnahme ist möglich, da der Anteil von Personen mit einem akademischen Abschluss in der Gruppe der Studienteilnehmenden höher als in der Bezugspopulation ist.

Die gewählte Abfrage der Versorgungsformen ermöglichte einen Vergleich zwischen den Pflegesettings – allerdings kann gleichzeitig nur eine indirekte Annahme darüber gemacht werden, welche Versorgungsform bevorzugt wird. Obwohl es sich um eine Querschnittsstudie handelt, können unter vorsichtiger Interpretation auch kausale Zusammenhänge unterstellt werden, da Präferenzen für eine zukünftig potenzielle eintretende pflegerische Versorgung erfragt wurden.

## Fazit für die Praxis

- Erwartungsgemäß zeigt sich eine hohe Präferenz für eine Pflege in der eigenen Häuslichkeit.
- Es lässt sich jedoch erkennen, dass eine häusliche Versorgung nicht originär mit informeller Pflege verbunden ist – meist wird eine professionelle Unterstützung gewünscht. Diese Erwartung indiziert zukünftig weiterhin einen hohen Bedarf an ambulanter Versorgung durch beruflich Pflegende.
- Überdurchschnittlich häufig favorisieren Personen ohne Berufsabschluss eine Pflege durch Angehörige. Auch Männer, Personen in einem Mehrpersonenhaushalt und Personen mit einer hohen eigenen Pflegebereitschaft präferieren häufiger diese Versorgungsform für die eigene Pflege.
